# Supplementary material for: Primary radiation damage in bone evolves via collagen destruction by photoelectrons and secondary emission self-absorption
Source: Nat Commun. 2022 Dec 20;13:7829. doi: 10.1038/s41467-022-34247-z (PMC9768145; doi:10.1038/s41467-022-34247-z)
Supplement: Supplementary file 1 — Supplementary Information [file 41467_2022_34247_MOESM1_ESM.pdf]

Supplementary Information

Primary radiation damage in bone evolves via collagen destruction by photoelectrons and secondary emission self-absorption

Katrein Sauer, Ivo Zizak, Jean-Baptiste Forien, Alexander Rack, Ernesto Scoppola, Paul Zaslansky

1 Supplementary Figures

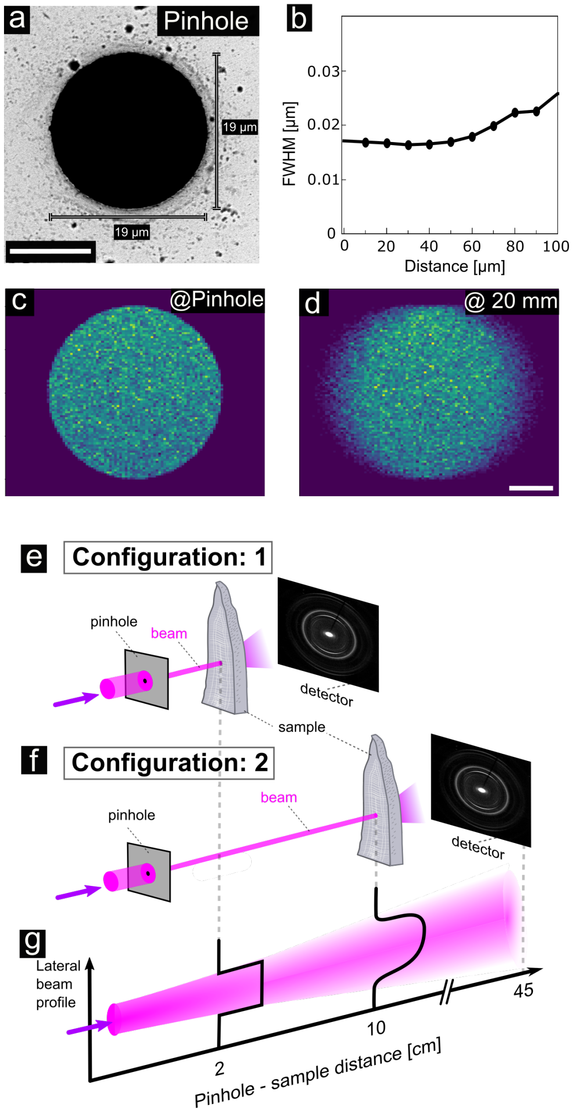

Supplementary Figure 1: ***Beamline and pinhole configurations.*** *For all 16 XRD experiments (Supplementary Table 1) we used four different pinholes to define the beam (Supplementary Table 2).* ***a*** *Back scattered scanning electron microscope image of the 20 μm sized pinhole. Scalebar: 10 μm. SEM-BEI pictures of all four pinholes used within this work look similar, and dimensions are listed in Supplementary Table 2.* ***b*** *Full-width half maximum (FWHM) of the horizontal beam diameter used in experiments on mySpot (BESSY, Helmholtz-Zentrum Berlin) based on ray-tracing simulations backed by experimental knife-edge transmission measurements: the FWHM changes minimally within the first few cm behind the ∼20 μm pinhole shown in (a). Simulations were performed for all four used beamsizes.* ***c*** *Simulated footprint of the X-ray at the pinhole* ***d*** *Simulated X-ray footprint 2 cm behind the pinhole. This is the distribution of photons impinging on the samples reported in the present study, identified below as 'configuration 1'. Simulation settings and details are provided in the Methods section in the main text.* ***e*** *Configuration 1 of the two configurations used for measurements: due to the short (2 cm) pinhole-to-sample distance, the beam has sharp edges at the sample position.* ***f*** *When the pinhole-to-sample distance is larger (10 cm) in configuration 2, the beam is blurred on the outer rims leading to the appearance of marked lateral tails at the sample position.* ***g*** *a schematic illustration of the mySpot mirror focused beam profile and gradual divergence as a function of increasing sample-to-pinhole distance.*

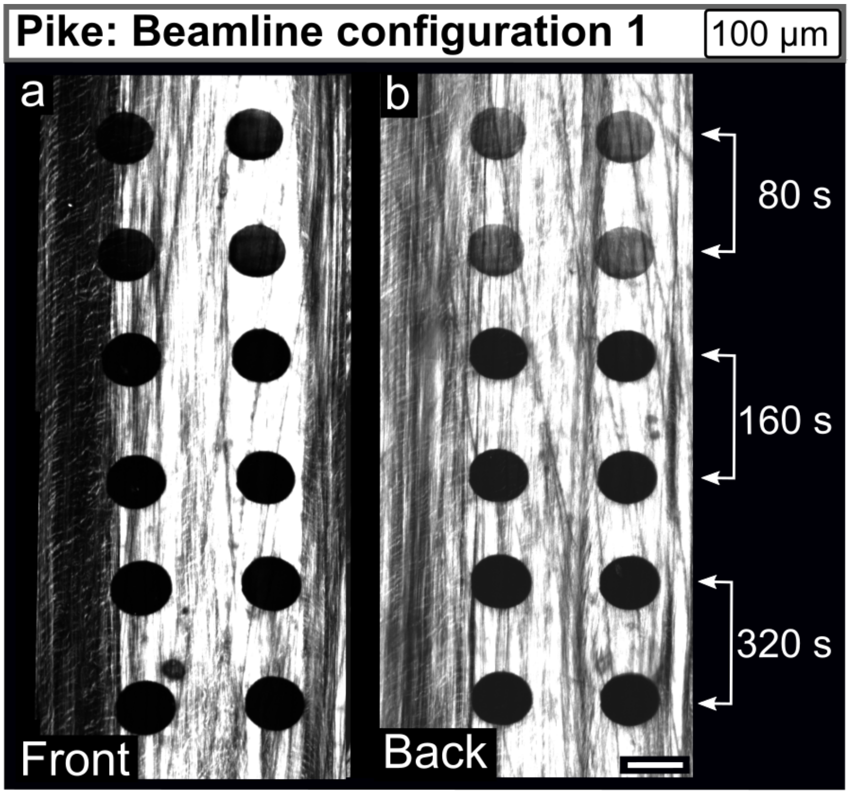

Supplementary Figure 2: ***Comparison of entry SHG images ('Front') and exit ('Back') irradiated sites on pike bone. a*** *Radiation damage observed on the 'Front' following different exposure times to X-rays with the* $\sim 100\,\mu\mathrm{m}$ *pinhole.* ***b*** *Radiation damage observed on the 'Back' of the same 300 µm thick sample shown in (a). White arrows identify the exposure times used. Due to the short pinhole to sample distance (configuration 1, Supplementary Figure 1e) damage spots at 80 s have sharp edges. The horizontal bone orientation has been flipped, matching 'Front' and 'Back' SHG imprints of damaged collagen. The damage reaches similar diameters in the 'Front' compared to the 'Back' as detailed in Supplementary Table 4. Regions in the 'Back' are partially protected from radiation damage compared to the regions in the 'Front', therefore, the damage observed on the 'Front' site is always more elaborate (n = 10). Scale bar: 100 µm.*

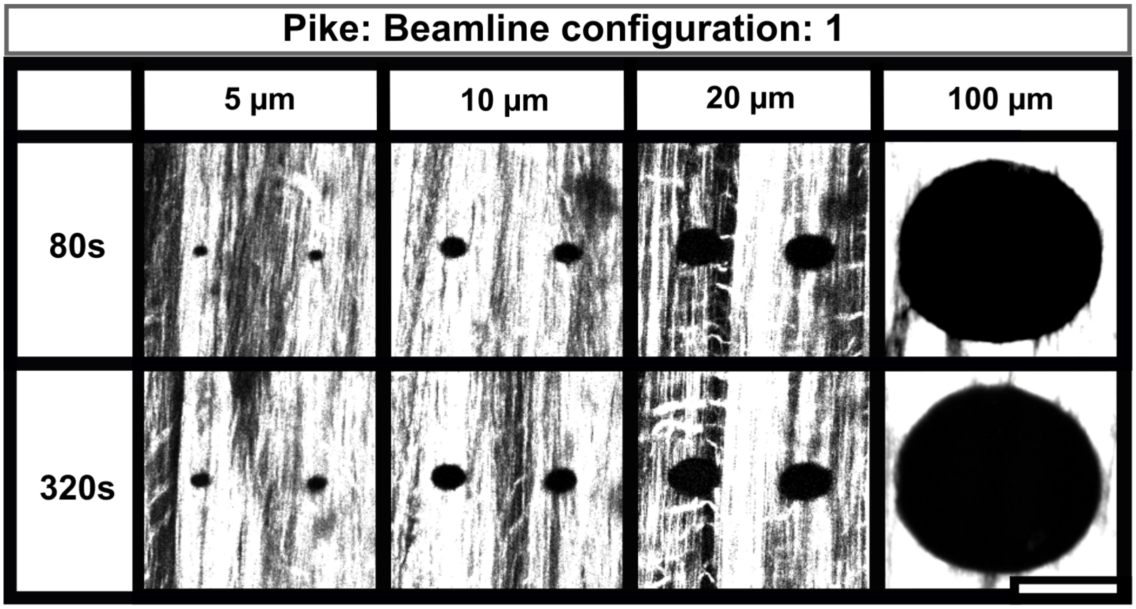

Supplementary Figure 3: ***Comparison of SHG images of different diameter beam footprints.*** *For all pinholes used (n = 4), damage at 320 s in pike bone is several micrometers larger than the damage observed at 80 s. Pinhole and damage sites are listed in Supplementary Table 2 and 4 (n = 10 samples). Scale bar: 50 µm. Contrast has been enhanced to highlight beam damage perimeter (compare Supplementary Figure 2).*

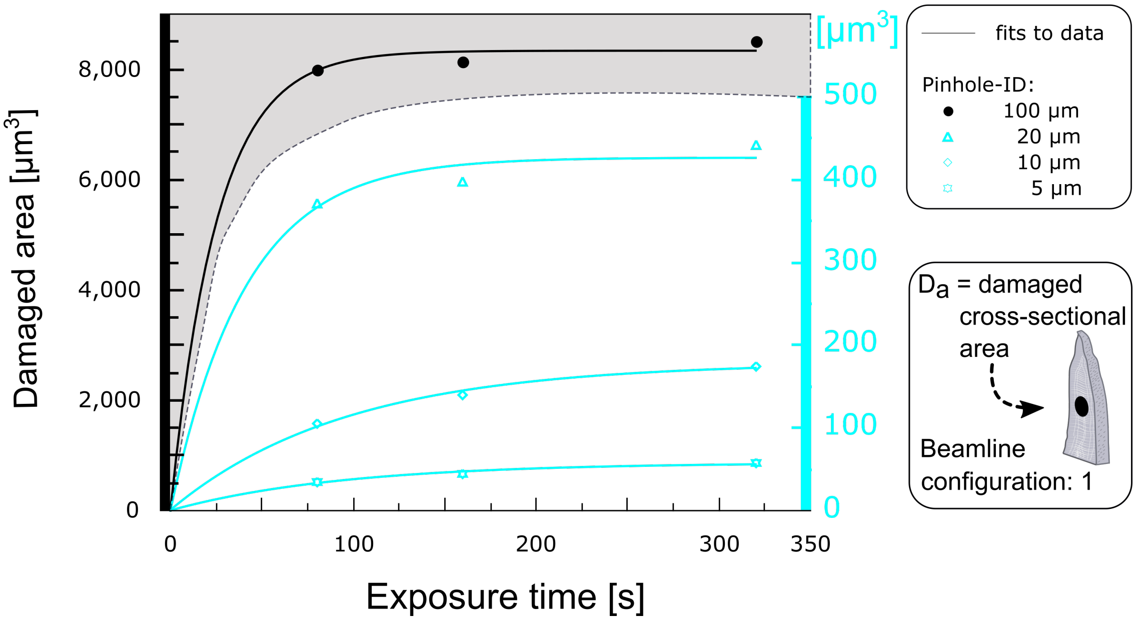

Supplementary Figure 4: ***Damaged area as a function of exposure time for different beam sizes.*** *Damage (D_a) observed using beamline configuration 1, as a function of exposure time for beam sizes of 5 µm, 10 µm, 20 µm, and 100 µm (Pinhole-ID's are listed in Supplementary Table 2) as observed on the outermost sample thickness visible by SHG (ca. 50 µm). Similar damage is visible on the 'Front' and the 'Back' for all samples irradiated for 80 s, 160 s and 320 s.*

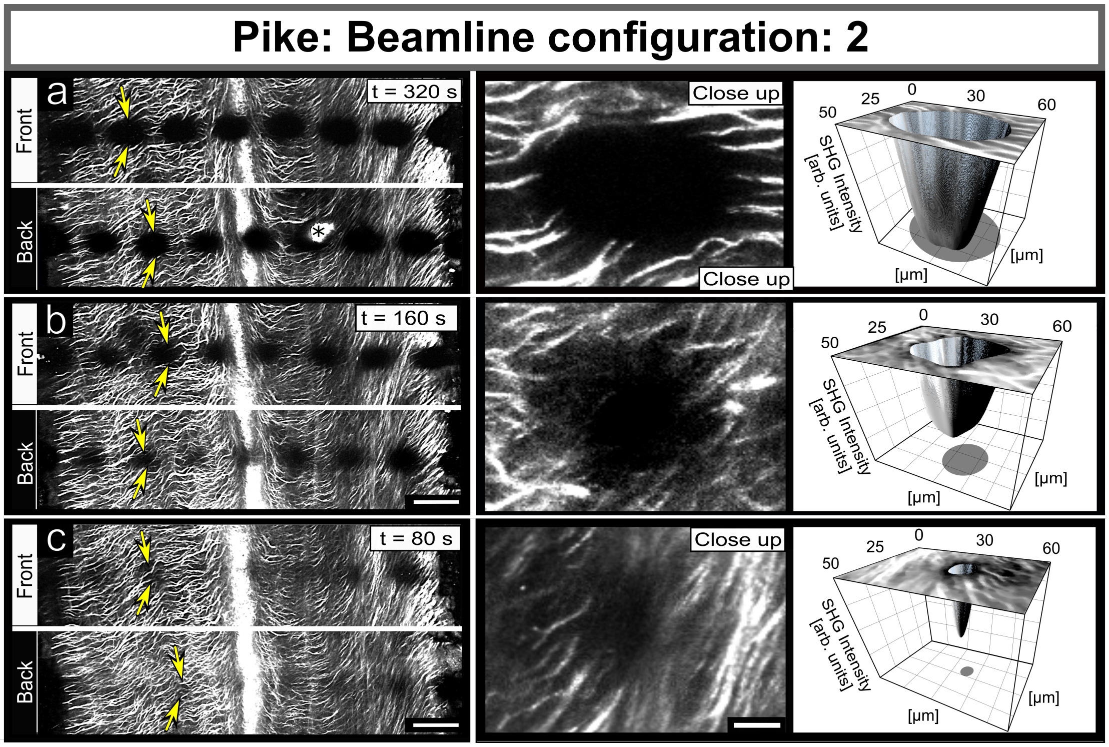

Supplementary Figure 5: ***Second harmonic generation (SHG) overview and close up images of bone exposed to X-ray diffraction (XRD) at larger distances from the pinhole.*** *Irradiating pike bone using beamline configuration 2 (Supplementary Fig. 1) exposed to different times as observed on X-ray beam entry ('Front') and exit sites ('Back') with typical close ups and pseudo-3D plots of the damaged area. These images demonstrate the effects of significant lateral beam tails and variable degrees of damage when using a beam devoid of sharp edges.* ***a*** *Bone exposed for 320 s. The SHG signal vanishes in sites where the beam damages collagen (black circular areas).* ***b*** *Bone irradiated for 160 s. The center is most affected.* ***c*** *Bone exposed for 80 s. A rather small area of dimming is revealed by the SHG signal in a narrow region in the center of X-ray illumination. Note that the images of the 'Back' samples have been flipped horizontally to facilitate comparison. The imprints of damage in the exit ('Back') are smaller than the imprints in the entry ('Front') sites in each case and for each point due to absorption in the sample. Yellow arrows highlight the damage for identical 'Front'-'Back' points. Overview scale bar is 50 µm. A black asterisk marks an artifact in one of the SHG measurement regions. Scale bar bar of close up images: 10 µm. Projections of the damaged zones are shown on the bottom of each pseudo-3D surface plot to indicate the lateral extent of radiation damage. Due to the large distance of pinhole and sample, beam divergence leads to smearing that is not observed in configuration 1 (Figure 2,3 and Supplementary Fig. 2 and 3).*

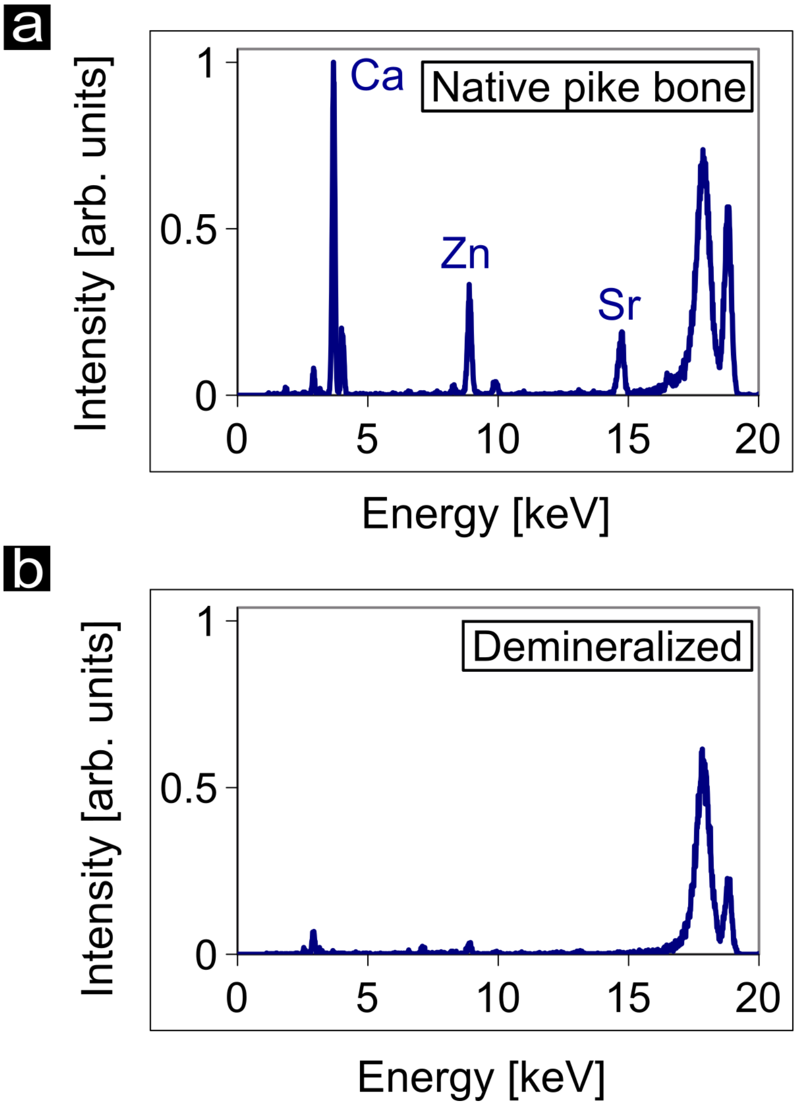

Supplementary Figure 6: ***Typical X-ray fluorescence (XRF) spectra of native and demineralized pike bone.*** ***a*** *XRF sum spectra of bone exhibit typical signatures: the prominent peak near 3.7 keV identifies calcium (Ca), the small peak near 2.9 keV is Ar in the air. Traces of zinc (Zn) and strontium (Sr) are also visible as well as ineleastic and elastic scatter of the incident 18 keV beam at energies >17 keV.* ***b*** *The fluorescence spectra of demineralized bone is practically devoid of Ca and Sr, showing only a trace of Zn. All measurements performed with an XRF silicon drift detector detector SiriusSD, SGX Sensortech* ($40\,\mathrm{mm}^2$).

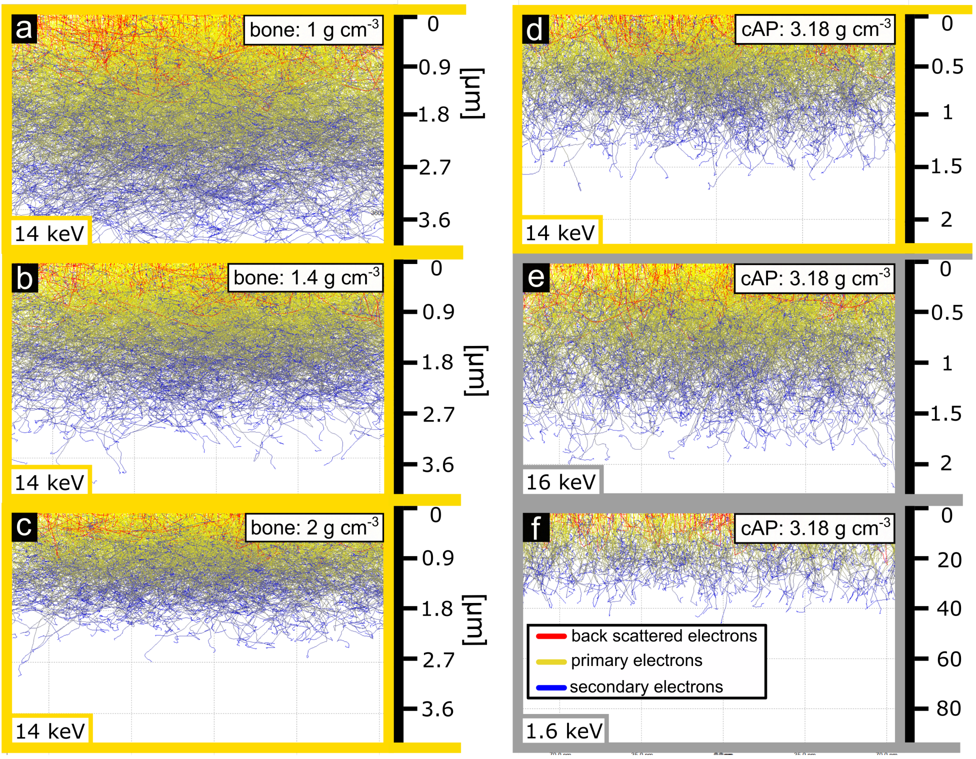

Supplementary Figure 7: ***Monte-Carlo simulations of electron trajectories through bone and carbonated apatite (cAP) exposed to electrons excited with different energies.*** *Due to X-ray irradiation of the cAP crystals and excitation of the Ca K-shell, many electrons will scatter across the bone with an energy difference of 18 keV - 4 keV ≈ 14 keV. Such electrons will interact with the surrounding matrix* ***a*** *up to ∼3.6 μm from the origin when the bone density is 1 g cm*$^{-3}$*,* ***b*** *up to ∼2.5 μm when the bone density is be 1.4 g cm*$^{-3}$ *and* ***c*** *up to ∼1.8 μm away from the source when the bone density comprises 2 g cm*$^{-3}$*.* ***d*** *Though strongly hindered in pure mineral in the cAP, such excited electrons will still propagate hundreds of nm away. Consequently, Ca K-shell electrons will always escape the nm sized cAP bony crystals in which they are formed, likely damaging the collagen surrounding them.* ***e*** *Electrons ejected from the K-shell of P (gray frame) by the high energy incoming X-rays of 18 keV carry an excess energy due to energy difference of 18 keV - 2 keV ≈ 16 keV. Such electrons will scatter further than the corresponding Ca K-shell electrons, as exemplified for simulations in pure cAP where they propagate about ∼1.5 μm. Although fewer in number (see Supplementary Notes 3.4) such electrons will scatter to greater distances when propagating across lower density bone regions (Supplementary Figure 8).* ***f*** *Radiation emitted due to Ca fluorescence (∼3.7 keV) will excite electrons in P (K-shell electron binding energy: ∼2.1 keV) that will propagate with an energy of 3.7 keV - 2.1 keV = 1.6 keV. Although lower than other interactions, even this energy is sufficient to propel electrons ∼20 nm or more through the mineral, thus likely emerging out of the 5 nm thick nanocrystals in which they are formed and ionizing the surrounding collagen fibers. All produced electrons are thus capable of breaking covalent bonds in the organic compartment that is always in close proximity to the mineral. Compositions for bone and mineral can be found in [1, 2].*

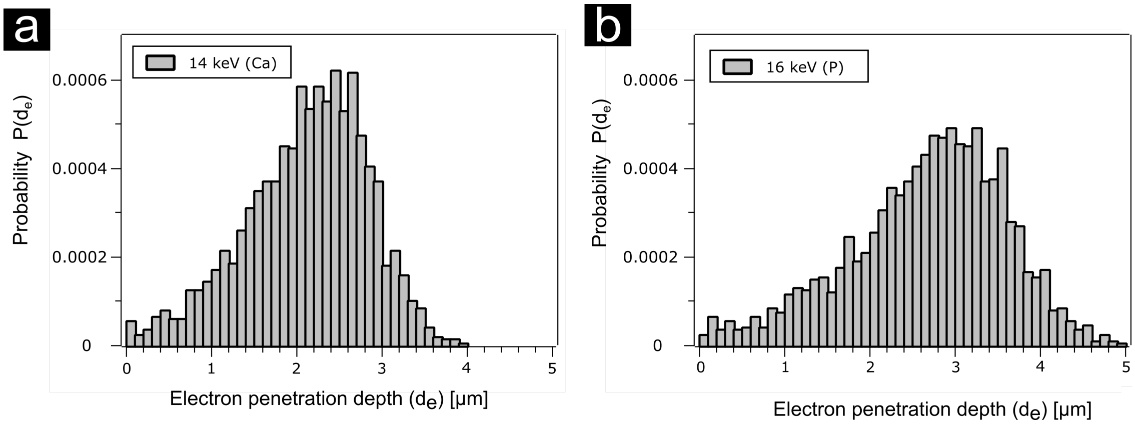

Supplementary Figure 8: ***Electron penetration depth distributions for different energies in bone.*** *Penetration depth probability distribution for* ***a*** *14 keV (calcium (Ca) K-shell) and* ***b*** *16 keV (phosphorous (P) K-shell) photoelectrons in bone specimens with bone compositions described in Supplementary Notes 3.1. Note that the higher energy 16 keV photoelectrons penetrate further in the bone matrix as compared to 14 keV photoelectrons. Data are obtained by Casino Monte Carlo simulations, described in the Methods section, as shown in Supplementary Figure 7.*

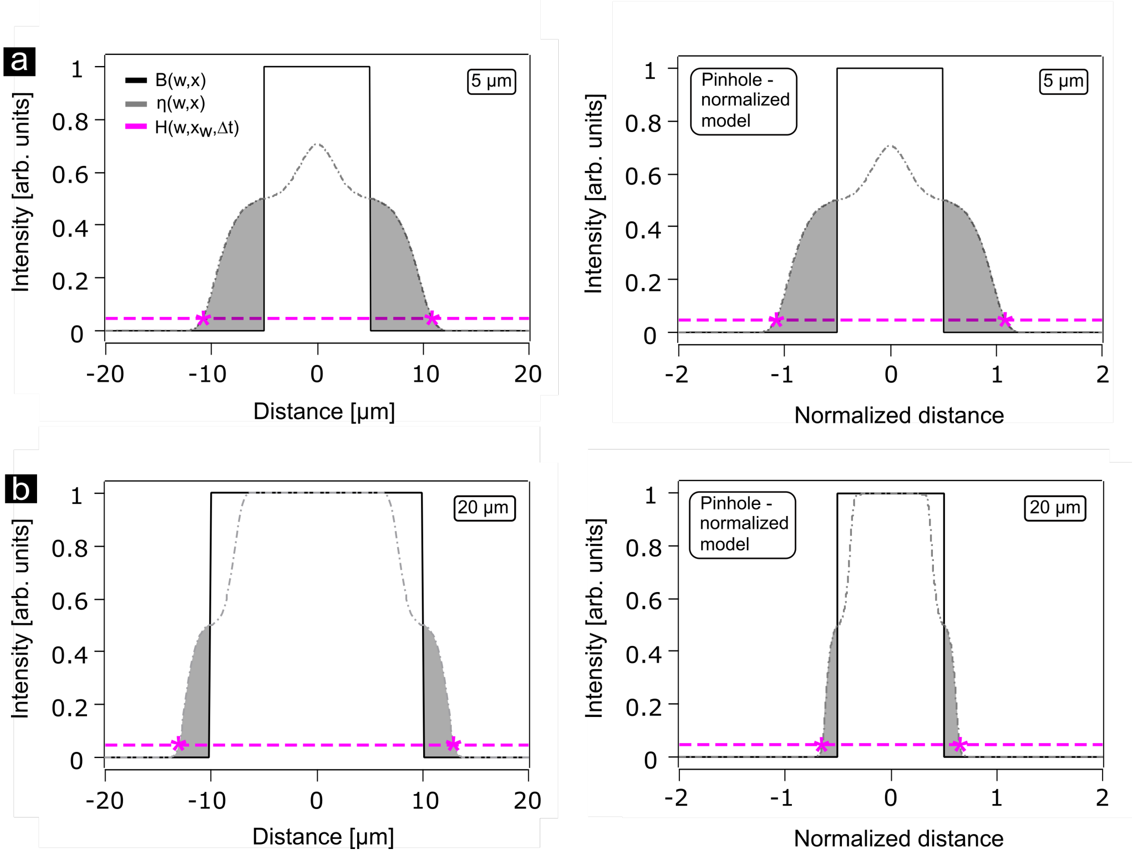

Supplementary Figure 9: ***Beam size dependant electron spread modeling.*** *Comparison between photoelectron source region $B(w,x)$ (continuous line, main text, Equation (2)) and energy deposition region $\eta(w,x)$ (dot-dash line, main text, Equation (3)) for* ***a*** *5 μm and* ***b*** *20 μm beam diameter. The domain in which electrons scatter outside of $B(w,x)$ is shaded to highlight the difference between the two. Pink dashed lines represent the intensity threshold $H(w,x,\Delta t)$=0.05, (main text Equation (5)), which intersects $\eta(w,x)$ for $x = x_w$ (pink stars), beyond which damage will be visible. In the pinhole-diameter normalized representation of each pinhole on the right, it is easier to observe the difference in magnitude of x_w highlighting the inverse relationship between the domain affected by electron scatter and the size of the source of electrons in irradiated bone. For this reason, the relative damaged volume increases with decreasing beam diameter.*

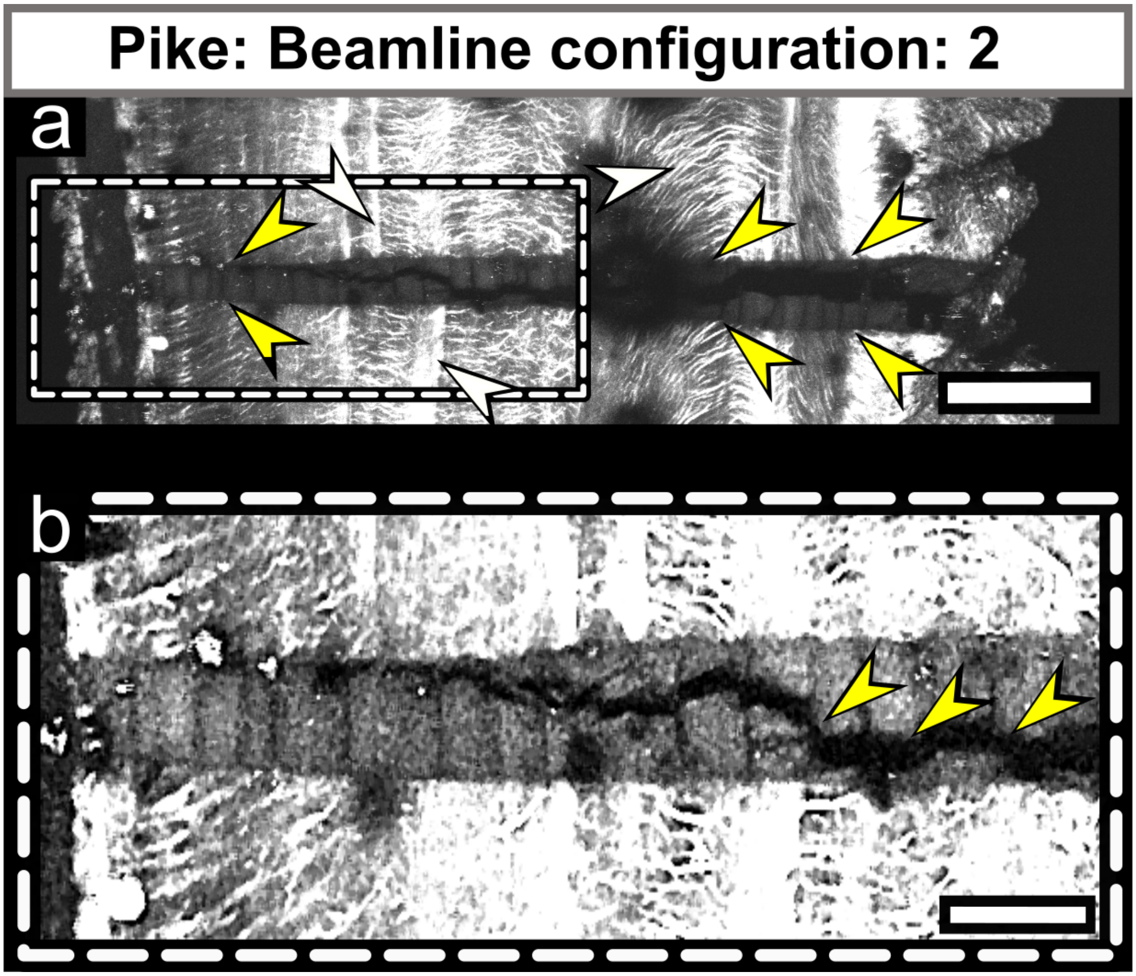

Supplementary Figure 10: ***Radiation-damage and cracking in pike bone scanned by XRD-μCT, magnified view of Figure 1c. a*** *SHG imaging reveals regions with intact collagen fibers in the bone sample (marked with white arrowheads). For XRD-μCT, the sample was repeatedly irradiated and in the damaged area, the collagen texture observed by SHG is lost. The results of radiation damage can be seen across the entire sample width (damage identified with yellow arrowheads). Scalebar: 150 μm.* ***b*** *Magnified, contrast-enhanced view of the area marked with dashed line in (a). A spontaneous crack with meandering path within the damaged region (marked with yellow arrows) propagates across the repeatedly irradiated area, attesting to damaged collagen and embrittlement due to accumulated damage. The sample was irradiated 43 times for 15 s each time. Scalebar: 50 μm.*

2 Supplementary Tables

Experiment	Dose [kGy]	E [keV]	I [ph/s]	t [s]	Beam area [µm^2]	pinhole [µm]
(1) XRD	14-113	18	$5.8 * 10^7$	40-320	$\pi \cdot 9.3 \cdot 9.7$	$20^{(c1)}$
(2) XRD	22-179	18	$6.6 * 10^6$	40-320	$\pi \cdot 3.1 \cdot 2.1$	$5^{(c1)}$
(3) XRD	17-136	18	$1.8 * 10^7$	40-320	$\pi \cdot 5.3 \cdot 4.4$	$10^{(c1)}$
(4) XRD	13-105	18	$5.4 * 10^7$	40-320	$\pi \cdot 9.3 \cdot 9.7$	$20^{(c1)}$
(5) XRD	10-40	18	$5.1 * 10^8$	80-320	$\pi \cdot 45.9 \cdot 48.4$	$100^{(c1)}$
(6) XRD	26-841	18	$1.0 * 10^9$	10-320	$\pi \cdot 16 \cdot 13$	$20^{(c2)}$
(7) XRD	39-2,367	18	$1.5 * 10^9$	10-600	$\pi \cdot 16 \cdot 13$	$20^{(c2)}$
(8) XRD	447-26,822	18	$1.7 * 10^{10}$	10-600	$\pi \cdot 16 \cdot 13$	$20^{(c2)}$
(9) XRD	9-1,041	18	$3.3 * 10^8$	10-1,200	$\pi \cdot 16 \cdot 13$	$20^{(c2)}$
(10) XRD	242-43,714	18	$4.0 * 10^9$	10-1,800	$\pi \cdot 9.3 \cdot 9.7$	$20^{(c1)}$
(11) XRD	109-32,785	18	$1.8 * 10^9$	10-3,000	$\pi \cdot 9.3 \cdot 9.7$	$20^{(c1)}$
(12) XRD	22-31,449	18	$3.7 * 10^8$	10-14,000	$\pi \cdot 9.3 \cdot 9.7$	$20^{(c1)}$
(13) XRD	546-98,355	18	$9.0 * 10^9$	10-1,800	$\pi \cdot 9.3 \cdot 9.7$	$20^{(c1)}$
(14) XRD	115-39,213	18	$1.9 * 10^9$	10-6,000	$\pi \cdot 9.3 \cdot 9.7$	$20^{(c1)}$
(15) XRD	118	18	$1.8 * 10^9$	25	$\pi \cdot 16 \cdot 13$	$20^{(c2)}$
(16) XRD-µCT	3,052	18	$1.8 * 10^9$	645	$\pi \cdot 16 \cdot 13$	$20^{(c2)}$
(17) µCT	2,689	34	$4.5 * 10^{11}$	960	400×1400	N/A
(18) µCT	8,854-70,833	35	$7.0 * 10^{12}$	240-1920	500×1400	N/A
(19) µCT	942-7,534	35	$5.6 * 10^{12}$	25-200	400×1400	N/A

Supplementary Table 1: ***Parameters of the synchrotron experiments included in this work for pike bones.*** *Summary of the main parameters used during the 19 synchrotron-based experiments with calculated radiation doses estimated using Equation (5) in (Supplementary Notes 3.2) according to [3]. For dose calculations of mass, the irradiated volume was considered based on knife-edge pinhole measurements at the sample position under consideration of beam broadening (see Supplementary Table 2). 'E' = the energy, 'I' = the intensity, 't' = the exposure time, and 'Beam area' = lateral incident beam size at sample position. Superscripts at pinhole sizes denote either configuration 1 (c1) or configuration 2 (c2) in Supplementary Figure 1 e or f, respectively.*

Pinhole-ID [µm]	SEM-BEI pinhole size [µm] ∅ (h)	∅ (v)	FWHM @2 cm [µm] ∅ (h)	∅ (v)	Beam size @2 cm [µm] ∅ (h)	∅ (v)
5	5.3	5.3	4.4	3.6	6.1	4.2
10	9.6	9.7	9.1	7.5	10.5	8.7
20	19.0	19.0	16.0	16.7	18.5	19.3
100	93.2	94.6	79.4	83.8	91.7	96.8

Supplementary Table 2: ***Beam diameter for different applied pinholes used with configuration 1*** *Geometric layout is depicted in Supplementary Figure 1e. Pinholes were imaged by SEM-BEI in which horizontal (h) and vertical (v) sizes were estimated for each pinhole-ID: of 5 µm, 10 µm, 20 µm, and 100 µm. To determine beam expansion 2 cm behind the pinhole, full width at half maximum (FWHM) of the derivatives of the intensity profiles measured in the beam path were extracted from knife-edge measurements at the sample position. Scaling the pinhole images by the ratio FWHM/sin(arccos(0.5)) we obtain the beam size 2 cm from the pinhole. These values correspond to simulations shown in Supplementary Figure 1.*

**20 µm pinhole**	DAMAGE SIZE [µm] **SHG**	**SEM**
320 s **(h)**	27.16 ± 0.72	27.77 ± 0.74
**(v)**	20.13 ± 0.41	22.76 ± 0.97
160 s **(h)**	25.51 ± 0.43	25.42 ± 1.41
**(v)**	19.28 ± 0.23	22.39 ± 0.84
80 s **(h)**	23.33 ± 1.18	25.07 ± 1.91
**(v)**	18.35 ± 0.27	21.46 ± 1.00
40 s **(h)**	21.40 ± 0.74	22.71 ± 2.32
**(v)**	17.49 ± 0.39	20.07 ± 2.06

Supplementary Table 3: ***Comparisons of damage determined by SHG and SEM-BEI images of irradiated pike bones.*** *Damage diameters following X-ray irradiation of 40 s, 80 s, 160 s, and 320 s when using mySpot configuration 1 and a 20 µm pinhole show no significant difference (t-test, p<0.05) between both methods. For all damage extents, horizontal (h) and vertical (v) beam diameters are shown. Values in light gray represent the standard deviations obtained for at least six measurements per method per exposure time.*

**Pinhole-ID [µm]**	**'FRONT' [µm]**	**'BACK' [µm]**
**5 (h)**	10.93 ± 0.49	10.33 ± 0.38
**(v)**	6.69 ± 0.93	6.45 ± 0.75
**10 (h)**	17.61 ± 0.93	17.64 ± 0.82
**(v)**	12.63 ± 0.45	11.66 ± 0.34
**20 (h)**	27.70 ± 0.60	27.46 ± 0.62
**(v)**	20.32 ± 0.36	19.54 ± 0.55
**100 (h)**	110.34 ± 0.85	110.04 ± 0.85
**(v)**	98.22 ± 0.69	98.03 ± 1.32

Supplementary Table 4: ***Collagen damage diameters on the incident X-ray beam entry site ('Front') and the corresponding exit site ('Back').*** *Due to the proximity of the pinhole and sample (beamline configuration 1) no difference is observed between 'Front' and 'Back' damage footprints for any of the examined beam sizes used (5 µm, 10 µm, 20 µm, and 100 µm pinhole). Measurements directly compare horizontal (h) and vertical (v) damage in SHG images following 320s exposure. Light gray values represent the standard deviations from six different damage site measurements.*

sample	40 s		320 s	
	∅ **(h)**	∅ **(v)**	∅ **(h)**	∅ **(v)**
pike cleithrum	21.40 ± 0.74	17.52 ± 0.39	27.16 ± 0.72	20.13 ± 0.41
pig jaw	17.67 ± 1.02	17.27 ± 0.73	22.62 ± 0.87	21.27 ± 0.38
mouse tibia	18.02 ± 0.57	17.99 ± 0.29	21.98 ± 0.42	20.22 ± 0.30
bovine tooth	18.79 ± 0.82	18.06 ± 0.53	21.18 ± 0.36	19.86 ± 0.81

Supplementary Table 5: ***Radiation damage diameters in different bony tissues, 20 μm pinhole.*** *SHG measurements of damage sites in multiple bone samples from different animals, comparing exposure to 40 s and 320 s. Pike exhibits approximately ∼15 % lateral enlargement horizontally (h) as compared with vertical (v) measurements.*

Beam diameter (w)	Relative damage $Z(w, \Delta t)$ following exposure for:		
[µm]	80 s	160 s	320 s
10	1.76	1.79	1.82
5	2.55	2.61	2.69
2	4.98	5.16	5.38
1	9.18	9.60	10.04
0.5	17.88	18.80	19.60
0.3	29.60	31.20	32.53
0.1	74.70	76.90	79.30

Supplementary Table 6: ***Predicted relative damage for decreasing beam sizes.*** *Values are calculated based on $Z(w, \Delta t)$ in equation (7) in the main text. Predictions are made for various beam diameters (1st column) and different exposure times (80 s, 120 s, 320 s).*

Species	Flux x 10^8 [ph/s]	Crystal size (Scherrer) [Å]	Crystal size (Voigt) [Å]	μ-strain fluct.	average c-lattice parameter [Å]
**Pike cleithrum**	3.3	205 ± 2	347 ± 25	0.0048 ± 0.0003	6.8740
	14.7	188 ± 1	324 ± 9	0.0052 ± 0.0001	6.8741
	170.0	191 ± 1	305 ± 3	0.0049 ± 0.00001	6.8756
**Bovine tooth**	3.7	237 ± 4	399 ± 29	0.0041 ± 0.0003	6.9155
	17.5	238 ± 3	392 ± 10	0.0041 ± 0.0001	6.9144
	40.0	242 ± 1	393 ± 6	0.0039 ± 0.0001	6.9150
**Mouse tibia**	3.7	249 ± 3	360 ± 11	0.0035 ± 0.0001	6.8764
	19.0	250 ± 1	351 ± 5	0.0034± 0.0001	6.8760
	90.0	249 ± 2	358 ± 4	0.0035 ± 0.0001	6.8759
**Pig jaw**	3.7	217 ± 6	344 ± 68	0.0043 ± 0.0007	6.8456
	19.0	214 ± 17	274 ± 146	0.0038 ± 0.036	6.8488
	90.0	211 ± 29	302 ± 71	0.0035 ± 0.001	6.8504

Supplementary Table 7: ***XRD characteristics and cAP nanocrystal attributes for samples measured by different fluxes in different bony samples from different animals.*** *Data are based on measurements of the (002) diffraction peak corresponding to the c-lattice parameter and include peak position and broadening analysis. Both the Scherrer equation and deconvolution by Voigt-fitting are used to obtain crystal sizes, the latter also revealing microstrain fluctuations according to [4].*

3 Supplementary Notes

3.1 Determination of bone density for dose calculations

In addition to producing scatter patterns, measurements of XRD in samples with known thickness provide direct information about transmission of radiation which can be used to obtain the density of the sample. If I and I_0 are beam intensities measured on and off the sample, respectively, transmission (T) is obtained from the ratio I/I_0, which in our samples was:

$$T = \frac{I}{I_0} = \frac{1.137 \times 10^9 ph/s}{1.47 \times 10^9 ph/s} \approx 0.75\,. \tag{1}$$

Transmission is related to sample thickness and absorption by the classical Beer–Lambert–Bouguer exponential relation $e^{-\mu d}$, where d is the sample thickness (in our case: 300 µm). We can obtain the linear attenuation coefficient μ of our samples directly by:

$$\mu = -\ln T/d = 9.6\ cm^{-1}\,, \tag{2}$$

and the reciprocal value, the attenuation length, is therefore:

$$d_{att} = 1/\mu = 1041\mu m\,. \tag{3}$$

Though the bone composition varies a bit, it is mainly the density that varies substantially within different bone samples [1]. For the fish bones used in this work, we assumed a reasonable approximated bone composition:

$$Ca_{(0.09863)}P_{(0.05918)}O_{(0.31413)}N_{(0.04946)}C_{(0.17861)}H_{(0.3)} \tag{4}$$

For an energy of 18 keV, which we used during all XRD measurements, d_{att} and various databases such as CXRO [5] can be used to obtain estimates for the pike bone mass density: $\rho_b = 1.4\,\mathrm{g\,cm^{-3}}$. Pike, similar to other fish, have low density as compared to other types of bone, and the corresponding mineral density (as reported elsewhere, [6]) is approximately $1\,\mathrm{g\,cm^{-3}}$.

3.2 Standard calculation of radiation dose absorbed in bone

To calculate the dose ($Dose$), we consider the energy (E), the flux (ϕ), the energy absorption (A_E) and the time (t), normalized by the mass (m) of the irradiated material, as often previously described [3]:

$$Dose = \frac{E \cdot \phi \cdot A_E \cdot t}{m}\ [Gray]. \tag{5}$$

The mass can be calculated from the density ($\rho_b = 1.4\,\mathrm{g\,cm^{-3}}$) and the irradiated volume, which is calculated from the sample thickness multiplied by the beam profile area (Supplementary Table 1: "Beam area [µm^2]"). Due to minimal divergence up to 10 cm from the pinhole (Supplementary Figure 1b), the horizontal and vertical beam dimensions are almost identical (Supplementary Table 2). In any single measurement point, we assume that the bone composition does not vary considerably and that the beam intensity was uniform and constant.

3.3 Mathematical description of damage expansion

We propose exponential fits to describe damage accumulation as visible in bone by SHG. Supplementary Figure 11 schematically depicts the accumulation of typical radiation damage with increasing exposure time. We consider first an infinitesimal small fragment of bone, irradiated by our X-ray beam (Supplementary Figure 11 a). At initial time $t = 0$, the sample (black framed object) is intact, and we turn on the photon beam (purple), which will irradiate the sample continuously. After an infinitesimally small time dt, the sample will have accumulated damage kdt (Supplementary Figure 11 b), where k is the percentage damage increase in unit time (% per second). When doubling the exposure time to $2dt$, the relative damage to the sample has grown to roughly $2kdt$ (Supplementary Figure 11 c).
Let V be the maximum damaged volume of the bone sample, which the beam can cause, after irradiation for infinite time (Supplementary Figure 11 f). At any earlier time (t), part of this volume will still be intact $N(t)$, whereas part of it will have been damaged by radiation $D(t)$. At time $t = 0$, when the beam is turned on, the entire sample is still intact, and we have $N(0) = V$ and $D(0) = 0$ (Supplementary Figure 11 d). At any later time t, we will always have

$$N(t) + D(t) = V. \tag{6}$$

The damage which accumulates between t and $(t+dt)$ is proportional to the intact sample volume $(N(t))$, can be expressed as (Supplementary Figure 11 e):

$$dD(t) = k \cdot N(t) \cdot dt, \tag{7}$$

from which we obtain:

$$\boxed{\frac{dD(t)}{dt} = k \cdot V - k \cdot D(t)} \tag{8}$$

By integrating and substitution, we obtain an exponential relation accounting for the total damage accumulation as a function of time (Supplementary Figure 11 f)):

$$\boxed{D(t) = V(1 - e^{-k \cdot t})} \tag{9}$$

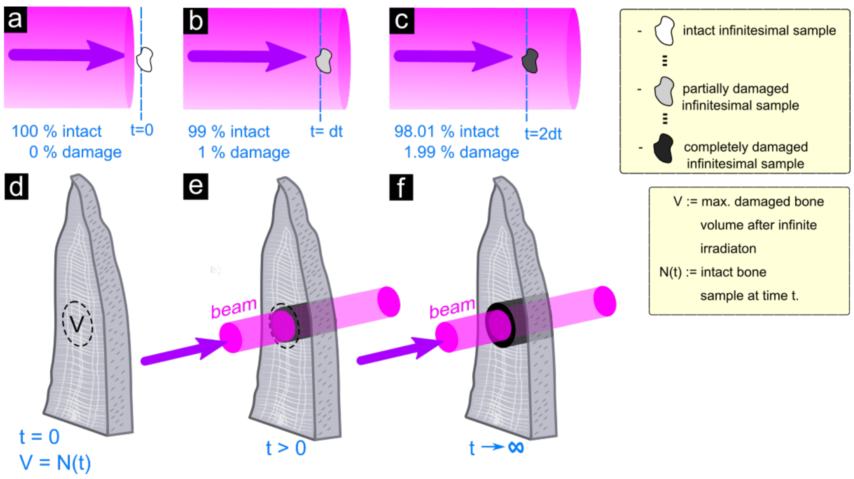

Supplementary Figure 11: ***Schematic representation of radiation damage accumulation.***

3.4 Absorption, radiation damage and cascades of scattering and fluorescence in bone

X-ray interactions such as excitation and ionization of bone nanocomposite ingredients occur via photoelectric absorption with simultaneous scattering (e.g., Compton scattering), fluorescence and emission of either photoelectrons or Auger electrons [7]. Some minor heating may also take place [8]. Ionizing radiation causes damage to bone by disrupting the collagen fibers which leads to embrittlement [9]. Radiation damage to bone increases with the dose [9, 10, 11, 12, 13] and has been investigated over many decades [14, 15, 16].

Exposure of bone to X-ray radiation will trigger a cascade of absorption and emission processes of both photons (fluorescence) and electrons (photoelectrons), the most relevant for this study being photoionization. These processes occur when the energy of the incident X-rays is higher than the electron binding energies of the heavier elements. The smaller the difference between the incident and binding energies, the higher the probability of interaction and photo absorption and as a consequence, photoelectron ejection. The probability for this to occur is known as photoionization cross section.

Depending on the incident X-ray photon energy, each element will have different absorption cross sections, which determine the portion of the incoming radiation absorbed. Since in bone all the core electron energies are lower than the energy of the incident beam, heavier elements absorb more than the lighter elements. This means, and calculations show that, for X-rays used in typical experiments, only a small percent of the energy delivered by the primary beam is directly absorbed by collagen. The main heavy elements in bone material are Ca and P, located mostly in the mineral cAP nanocrystals, and once they absorb photons following exposure to hard (>5 keV) X-rays, photoelectrons will be emitted. The resulting photoelectrons have energies spanning a range of several keV, and they therefore scatter through the sample, ionizing and inducing radiation damage in adjacent regions. Even the lower energies of secondary excitations such as electrons excited in P inside the nanocrystals due to Ca X-ray fluorescence (XRF) are sufficient to scatter electrons across tens of nm which means that they will emerge out of the mineral phase (See Monte Carlo simulations, Supplementary Figure 7).

For a given incident X-ray energy E and electron binding energy BE, the number of photoionized atoms and corresponding ejected photoelectrons $n_k^{(e)}(E-BE)$ from a sample of thickness d, irradiated by and X-ray beam of flux $I_0(E)$, can be calculated by considering the photoionization cross section $\sigma_k^{(ion.)}(BE, E)$ of the k-th element (in cm^2 g^{-1}) [17] and the local density ρ_k (g cm^{-3}) as:

$$n_k^{(e)}(E-BE) = I_0(E) \cdot \sigma_k^{(ion.)}(BE, E) \cdot \rho_k \cdot d, \tag{10}$$

where the incident photon of energy E is absorbed and the energy corresponding to the binding energy of the ejected photoelectron is deposited. The remaining energy difference $(E-BE)$ is transferred to the scattered photoelectron. Subsequently, several processes might occur to fill the lacuna generated by the ejected electron in the excited element electron shells. One outcome will be relaxation of electronic clouds generating the emission of XRF photons. The amount of generated photons $n_{k,\Gamma}^{(XRF)}(E')$ from a given transition Γ (for instance KL3, KL2 etc.) [18] with a given energy E' can be calculated:

$$n_{k,\Gamma}^{(XRF)}(E') = n_k^{(e)}(E-BE) \cdot \phi_{k,\Gamma} \cdot r_{k,\Gamma} \tag{11}$$

with $\phi_{k,\Gamma}$ and $r_{k,\Gamma}$ corresponding to the fluorescence yield and the radiative rate, respectively. While the fluorescence yield depends on the empty electronic shell that is filled after relaxation, the radiative rate depends on both the intial and final shells of the transitioning electron. Therefore, the fluorescence yield is equal for all the K (or the L, M) transitions of the k-th element and the radiative rate is different for all the transitions (KL3, KL2, L1L2, etc.). The emitted fluorescence photons will carry an energy E' equal to the energy difference of the shells involved in the relaxation process. A typical XRF spectrum of pike bone is shown in Supplementary Figure 6a showing a clear peak for Ca-K shell fluorescence, that is totally missing in demineralized pike bone, Supplementary Figure 6b. P cannot be seen due to absorption of the signal on the path to the XRF detector.

Emitted particles (i.e. photoelectrons and XRF photons) will thus always form when X-rays interact with cAP crystals in bony specimens. We consider a bone composition as presented in (4) (ρ_{Ca} ≈0.41 g cm^{-3}, ρ_P ≈0.19 g cm^{-3}) and the corresponding values of $\sigma_k^{(ion.)}(BE, E)$ for the previously mentioned atoms. The energy carried by ejected photoelectrons $E_k^{photoel}$ and converted to Joule (1 eV ≈ 1.6·10^{-19} J):

$$E_k^{photoel.} = n_k^{(e)}(E - BE) \cdot (E - BE) \cdot 1.6 \cdot 10^{-19} J. \tag{12}$$

From this, we calculate the ratio of energies carried by the P and Ca photoelectrons ejected by their K-shells when interacting with 18 keV incident photons:

$$\begin{aligned}
\frac{E_P^{photoel.}}{E_{Ca}^{photoel.}} &= \frac{n_P^{(e)}(18 - 2.1455) \cdot (18 - 2.1455)}{n_{Ca}^{(e)}(18 - 4.0381) \cdot (18 - 4.0381)} \\
&= \frac{\sigma_P^{(ion.)}(2.1455, 18) \cdot \rho_P \cdot (18 - 2.1455)}{\sigma_{Ca}^{(ion.)}(4.0381, 18) \cdot \rho_{Ca} \cdot (18 - 4.0381)} \\
&\approx \frac{6.31851 \cdot 0.19 \cdot (18 - 2.1455)}{15.5272 \cdot 0.41 \cdot (18 - 4.0381)} \\
&\approx 0.22.
\end{aligned} \tag{13}$$

P photoelectrons ejected by the incident beam from the K-shell thus carry approximately 1/5 of the energy carried by the abundant Ca K-shell photoelectrons. P K-shell photoelectrons have higher energy (≈ 16 keV) as compared to Ca K-shell photoelectrons (≈ 14 keV) and they can therefore scatter to greater distances in the bone specimen, as shown by simulations in Supplementary Figs 7 and 8. Nevertheless, most of the electrons scattered into the surrounding bone material are due to electron excitation in Ca from the incoming incident beam. It is for this reason, that there is such a good fit between experiment and prediction, shown in Figure 4 of the main text. The same calculation can be performed to estimate the energy ratio between KL3 (most abundant) XRF photons and K-shell photoelectrons emitted by Ca, using Equations (10, 11) and similar energy conversions as shown by Equation (12):

$$\begin{aligned}
\frac{E_{Ca}^{XRF}}{E_{Ca}^{photoel.}} &= \frac{n_{Ca,KL3}^{(XRF)}(3.6881) \cdot 3.6881}{n_{Ca}^{(e)}(18 - 4.0381) \cdot (18 - 4.0381)} \\
&= \frac{n_{Ca}^{(e)}(18 - 4.0381) \cdot \phi_{Ca,\mathrm{KL3}} \cdot r_{Ca,\mathrm{KL3}} \cdot 3.6881}{n_{Ca}^{(e)}(18 - 4.0381) \cdot (18 - 4.0381)} \\
&= \frac{\phi_{Ca,\mathrm{KL3}} \cdot r_{Ca,\mathrm{KL3}} \cdot 3.6881}{18 - 4.0381} \\
&= \frac{0.1687 \cdot 0.58992 \cdot 3.6881}{18 - 4.0381} \\
&\approx 0.03.
\end{aligned} \tag{14}$$

Regardless of their penetration depth, Equation (14) shows that Ca KL3 XRF photons carry a negligible amount of energy with respect to Ca K-shell photoelectrons. Therefore, the latter deposit far more energy into the specimen causing significant ionization.

Supplementary References

[1] Johns, H. E. & Cunningham, J. R. The Physics Of Radiology. *Charles C Thomas, Springfield, Illinois, USA* **4th Edn edition**, (1983).

[2] Zapanta LeGeros, R. Apatites in biological systems. *Prog. Cryst. Growth Charact.* **4**, 1-45 (1981).

[3] Deymier-Black, A. C., Almer, J. D., Stock, S. R. & Dunand, D. C. Variability in the elastic properties of bovine dentin at multiple length scales. *J. Mech. Behav. Biomed. Mater.* **5**, 71-81 (2012).

[4] Emil Zolotoyabko Basic Concepts Of X-Ray Diffraction. *John Wiley & Sons, Weinheim, Germany.* , (2014).

[5] Henke, B. L., Gullikson, E. M. & Davis, J. C. X-ray interactions: photoabsorption, scattering, transmission, and reflection at E=50-30000 eV, Z=1-92. *Atomic Data and Nuclear Data Tables* **54**, https://henke.lbl.gov/optical_constants/atten2.html (1993).

[6] Atkins, A. *et al.* Remodeling in bone without osteocytes: Billfish challenge bone structure–function paradigms. *PNAS* **111**, 16047-16052 (2014).

[7] Nave, C. Radiation damage in protein crystallography. *Radiation Physics and Chemistry* **45**, 483-490, (1995).

[8] Garman, E. F. & Weik, M. X-ray radiation damage to biological samples: recent progress. *J. Synchrotron Radiat.* **26**, 907-911 (2019).

[9] Currey, J. D. *et al.* Effects of ionizing radiation on the mechanical properties of human bone. *J. Orthop. Res.* **15**, 111-117 (1997).

[10] Blake, C. & Phillips, D. C. Proceedings of the Symposium on the Biological Effects of Ionizing Radiation at the Molecular Level. *International Atomic Energy Agency Vienna* https://nla.gov.au/nla.cat-vn2475863 , 183-191 (1962).

[11] Smith, C. W., Young, I. S. & Kearney, J. N. Mechanical properties of tendons: changes with sterilization and preservation. *J. Biomech. Eng.* **118**, 56—61 (1996).

[12] Cornu, O., Banse, X., Docquier, P. L., Luyckx, S. & Delloye, Ch. Effect of freeze-drying and gamma irradiation on the mechanical properties of human cancellous bone. *J. Orthop. Res.* **18**, 426-431 (2000).

[13] Teng, T.-Y. & Moffat, K. Primary radiation damage of protein crystals by an intense synchrotron X-ray beam. *J. Synchrotron Radiat.* **7**, 313-317 (2000).

[14] Bowes, J. H. & Moss, J. A. The effect of gamma irradiation on collagen. *Radiat. Res.* **16**, 211-223 (1962).

[15] Dziedzic-Goclawska, A., Kaminski, A., Uhrynowska-Tyszkiewicz, I. & Stachowicz, W. Irradiation as a safety procedure in tissue banking. *Cell Tissue Bank.* **6**, 201-219 (2005).

[16] Gouk, S.-S., Lim, T.-M., Teoh, S.-H. & Sun, W. Q. Alterations of human acellular tissue matrix by gamma irradiation: Histology, biomechanical property, stability, in vitro cell repopulation, and remodeling. *J. Biomed. Mater. Res. Part B Appl. Biomater.* **84B**, 205-217 (2008).

[17] Tom Schoonjans, T. *et al.* The xraylib library for X-ray–matter interactions. Recent developments. *Spectrochimica Acta Part B: Atomic Spectroscopy* **66**, 776-784, (2011).

[18] Beckhoff, B., Kanngießer, B., Langhoff, N., Wedell, R. & Wolff, H. Handbook of practical X-ray fluorescence analysis. *Springer* (2006).
