# Supplementary material for: Primary radiation damage in bone evolves via collagen destruction by photoelectrons and secondary emission self-absorption
Source: Nat Commun. 2022 Dec 20;13:7829. doi: 10.1038/s41467-022-34247-z (PMC9768145; doi:10.1038/s41467-022-34247-z)
Supplement: Supplementary file 3 — Reporting Summary [file 41467_2022_34247_MOESM3_ESM.pdf]

nature portfolio

Corresponding author(s): Katrein Sauer, NCOMMS-21-46473

Last updated by author(s): Oct 5, 2022

Reporting Summary

Nature Portfolio wishes to improve the reproducibility of the work that we publish. This form provides structure for consistency and transparency in reporting. For further information on Nature Portfolio policies, see our Editorial Policies and the Editorial Policy Checklist.

Statistics

For all statistical analyses, confirm that the following items are present in the figure legend, table legend, main text, or Methods section.

n/a	Confirmed	
☐	☒	The exact sample size (*n*) for each experimental group/condition, given as a discrete number and unit of measurement
☐	☒	A statement on whether measurements were taken from distinct samples or whether the same sample was measured repeatedly
☐	☒	The statistical test(s) used AND whether they are one- or two-sided<br>*Only common tests should be described solely by name; describe more complex techniques in the Methods section.*
☒	☐	A description of all covariates tested
☒	☐	A description of any assumptions or corrections, such as tests of normality and adjustment for multiple comparisons
☒	☐	A full description of the statistical parameters including central tendency (e.g. means) or other basic estimates (e.g. regression coefficient) AND variation (e.g. standard deviation) or associated estimates of uncertainty (e.g. confidence intervals)
☒	☐	For null hypothesis testing, the test statistic (e.g. *F*, *t*, *r*) with confidence intervals, effect sizes, degrees of freedom and *P* value noted<br>*Give P values as exact values whenever suitable.*
☐	☐	For Bayesian analysis, information on the choice of priors and Markov chain Monte Carlo settings
☐	☐	For hierarchical and complex designs, identification of the appropriate level for tests and full reporting of outcomes
☐	☐	Estimates of effect sizes (e.g. Cohen's *d*, Pearson's *r*), indicating how they were calculated

Our web collection on statistics for biologists contains articles on many of the points above.

Software and code

Policy information about availability of computer code

Data collection	Leica Application Suite Advanced Fluorescence Lite (LAS AF Lite) V2.6.0 build 7266, Dectris Eiger Albula software beamline custom code collected via spec, SEM phenom user interface version 1.7, ESRF pyhst2
Data analysis	Quantification of SHG images: Fiji Version 1.52i, XRD analysis with XRDUA software package (version 6.4.3.2), Diffraction profiles were fitted using Python 3.7 and the LMFIT package. Monte Carlo simulations were performed with the Casino modeling tool v2.51 (2.5.1.0). Graphs were produced in QtiPlot v0.9.8.8 svn 255 and Figures were produced in Inkscape v0.92 and POV-Ray v3.7. To calculate the divergence at sample position the BESSY Ray-UI: User interface (ray tracing software) for ray was used. Python v3.7 was used for the evaluation of equation (6) and (7).

For manuscripts utilizing custom algorithms or software that are central to the research but not yet described in published literature, software must be made available to editors and reviewers. We strongly encourage code deposition in a community repository (e.g. GitHub). See the Nature Portfolio guidelines for submitting code & software for further information.

Data

Policy information about availability of data

All manuscripts must include a data availability statement. This statement should provide the following information, where applicable:

- Accession codes, unique identifiers, or web links for publicly available datasets
- A description of any restrictions on data availability
- For clinical datasets or third party data, please ensure that the statement adheres to our policy

The datasets generated during and/or analysed during the current study are available from the corresponding author on reasonable request.

Human research participants

Policy information about studies involving human research participants and Sex and Gender in Research.

Reporting on sex and gender	N/A
Population characteristics	N/A
Recruitment	N/A
Ethics oversight	N/A

Note that full information on the approval of the study protocol must also be provided in the manuscript.

Field-specific reporting

Please select the one below that is the best fit for your research. If you are not sure, read the appropriate sections before making your selection.

[x] Life sciences [ ] Behavioural & social sciences [ ] Ecological, evolutionary & environmental sciences

For a reference copy of the document with all sections, see nature.com/documents/nr-reporting-summary-flat.pdf

Life sciences study design

All studies must disclose on these points even when the disclosure is negative.

Sample size	The main findings of the present study were discovered and worked out in fish bones exposed to X-rays in tomography and XRD experiments. A total of 64 fish bone samples were used in different radiation situation to observe and quantify radiation damage. Once we were certain about damage in these bones, additional confirmatory experiments were performed in 3 pig jaw segments, 2 bovine teeth and 1 mouse bone. Damage was visible in every exposure beyond the threshold we determined. Therefore, a large sample size was used for fish whereas only representative samples were used as proof of principle of damage evoluion across different mineralized tissues.
Data exclusions	Some of the XRD data were excluded due to beam loss or sample missalignment. Due to a large number of samples measured by SHG, and to ease quantification, only a subset of the data was used were all margins of damage were clearly visible avoiding imaging shadows and compromised contrast or unclear collagen microstructure. In the smaller fish bone samples, at least 10 measurement points were taken per experimental condition (exposure time, beam size, etc.). The other bone samples were larger and therefore the number of irradiation points was larger.
Replication	All spots in bone radiated more than 40 s exhibited damage in every case. We succesfully replicated damage in different bone locations, different beamsizes, different fluxes and different bone types.
Randomization	We used a large number of different bone pieces of different animals collected from different sources (cleithrum of pikes, mouse tibia, bovine tooth and pig jaw). There was no need for random allocation in this study, simply because in ever bone tested we were looking for damage that always emerged.
Blinding	In this study we examined the interactions between photons, electrons and the nanocomposite of bone. This does not require any blinding.

Reporting for specific materials, systems and methods

We require information from authors about some types of materials, experimental systems and methods used in many studies. Here, indicate whether each material, system or method listed is relevant to your study. If you are not sure if a list item applies to your research, read the appropriate section before selecting a response.

Materials & experimental systems

n/a	Involved in the study
☒	☐ Antibodies
☒	☐ Eukaryotic cell lines
☒	☐ Palaeontology and archaeology
☒	☐ Animals and other organisms
☒	☐ Clinical data
☒	☐ Dual use research of concern

Methods

n/a	Involved in the study
☒	☐ ChIP-seq
☒	☐ Flow cytometry
☒	☐ MRI-based neuroimaging
